# Information Wanted

**Published:** 1913-06

**Authors:** 


### Information Wanted

In a case of DELIRIUM TREMENS, the vomitus was negative as to blood, peptone, sugar and HCl. The total acidity by phenophthalein was 15 degrees. The alizarin end-point was not reached at 120 degrees. Now, while the alizarin test is very inaccurate, it is of interest to have an explanation of the strange behavior at times.

